# Circulating Cell-Free Tumour DNA in the Management of Cancer

**DOI:** 10.3390/ijms160614122

**Published:** 2015-06-19

**Authors:** Glenn Francis, Sandra Stein

**Affiliations:** 1 Director Pathology, Genomics for Life, Herston 4006, Australia; 2 School of Medicine, Griffith University, Gold Coast 4215, Australia; 3 Australian Institute for Bioengineering and Nanotechnology, University of Queensland, St Lucia 4067, Australia; 4 Laboratory Director, Genomics for Life, Herston 4006, Australia; E-Mail: srstein@genomicsforlife.com.au

**Keywords:** cell-free DNA, cell-free tumour DNA, non-small cell lung cancer, melanoma, colorectal carcinoma, minimal residual disease, liquid biopsy

## Abstract

With the development of new sensitive molecular techniques, circulating cell-free tumour DNA containing mutations can be identified in the plasma of cancer patients. The applications of this technology may result in significant changes to the care and management of cancer patients. Whilst, currently, these “liquid biopsies” are used to supplement the histological diagnosis of cancer and metastatic disease, in the future these assays may replace the need for invasive procedures. Applications include the monitoring of tumour burden, the monitoring of minimal residual disease, monitoring of tumour heterogeneity, monitoring of molecular resistance and early diagnosis of tumours and metastatic disease.

## 1. Introduction

A biomarker is a chemical or biological compound that can be used to diagnose or monitor disease. For cancer, the aims of a sensitive biomarker are to enable early detection of cancer, monitoring of disease progression and response to treatment. Biomarkers can be broadly grouped into predictive biomarkers and prognostic biomarkers. A predictive factor is defined as a clinical, pathologic or clinical feature that determines the likelihood of a response to a particular treatment. A prognostic factor is defined as a clinical, pathologic or clinical biomarker that determines patient outcome or survival. A biomarker may be both predictive and prognostic. For a factor to be useful, it must be technically validated, clinically validated and influence clinical decision making [1].

The first cancer biomarker was described in 1848, a study identified the presence immunoglobulin light chains in urine in 75% of patients with multiple myeloma [2]. This cancer biomarker is still in use today, although laboratory techniques have improved the ability to more accurately quantify this biomarker.

During the 1900s a number of chemicals (proteins, enzymes and hormones) were identified in biological fluids from cancer patients, however monitoring of malignant diseases essentially started with the identification of two biomarkers (alpha-fetoprotein and carcinoembryonic antigen) in the 1960s. Originally the assays for these biomarkers used radioisotopes but this technology was replaced with enzyme immunoassay methods in the 1980s.

Biomarker discovery in cancer has rapidly proliferated and thousands of biomarkers have been described, but relatively few are in clinical use. The failure to translate the biomarker into clinical practice may relate to technical challenges in the testing itself in some cases, but in most cases it is usually due to overlap of ranges between normal and cancer patients so that normal and cancer patients cannot be clearly separated from each other. Most biomarkers show a relative difference in expression between normal and cancers cells with increased levels of expression in cancer cells. Even protein biomarkers that are in clinical use such as CA15.2, CA19.9, CA125 and PSA that are used to monitor response to treatment and in some cases for early detection of malignancy, suffer from a lack of sensitivity and specificity and do not always show good concordance with tumour stage [3].

Different modalities such as biomarkers and imaging studies are often combined to evaluate cancer progression, but the increasing number of treatment options requires a more efficient evaluation of tumour response to enable optimisation of treatment decisions.

Biomarkers may be detected in various bodily fluids such as blood, urine and sputum or in the tumour tissues themselves. A diagnosis of cancer is usually made based on the morphological assessment of tumour tissue obtained from a biopsy, surgical excision or fine-needle aspirate. These are relatively invasive procedures and in some circumstances there may be limited tissue obtained or the tumour may not be amenable to biopsy or excision precluding the determination of a clinically useful biomarker. Concurrently with the search for new clinically relevant biomarkers, the search has also been for relatively non-invasive patient-friendly biomarkers to enable frequent monitoring of cancer patients with a relatively low cost.

## 2. Molecular Biomarkers in Tissue

The completion of the Human Genome Project has expanded the field of biomarkers to now include DNA, RNA, ncRNA, miRNA and epigenetics. International collaborative projects including the International Cancer Genome Consortium and the Catalogue of Mutations in Cancer (COSMIC) have identified driver mutations in multiple cancer types. Some of these molecular biomarkers are now in routine clinical practice. These include BRAF mutation analysis for melanoma, EGFR and ALK testing for lung cancer and RAS testing for colorectal cancer.

## 3. Melanoma

Mutations in BRAF, GNA11, GNAQ, KIT, MEK1 (MAP2K1), and NRAS can be found in approximately 70% of all melanomas. Mutations in the BRAF gene occur in approximately 48% of metastatic melanomas [4]. In melanoma the majority of mutations result in a substitution at the V600 protein in the activating portion of the tyrosine kinase. The majority of the BRAF V600 mutations are V600E (*BRAF* c.1799T>A) resulting in increased activity of the tyrosine kinase [4]. The clinical significance of this is that there are targeted therapies that are more effective in tumours with the activating mutations than wild type tumours. The determination of BRAF mutation status is therefore critical when evaluating a patient for anti-BRAF targeted therapy and has been implemented into routine clinical practice. For melanoma, the activating V600 mutations, including V600E, are associated with increased sensitivity to BRAF inhibitors such as vemurafenib and dabrafenib [5,6,7]. Recent clinical trials have also shown that combination therapy using dabrafenib and trametinib has shown an improved overall survival compared to dabrafenib alone [6] and combination therapy using vemurafenib and cobimetinib has also shown improved survival compared to vemurafenib alone [7].

## 4. Non-Small Cell Lung Cancer

In 2012, Lung cancer was the 5th most commonly reported cancer in Australia with 11,280 reported cases. In 2012 lung cancer was the most common cause of cancer death with 8099 people dying due to this disease [8]. Lung cancer has one of the lowest reported survival rates of 14% from 2006–2010 [9].

Lung cancer is divided into small cell lung cancer comprising approximately 15% of cases and non-small cell lung cancer (NSCLC). NSCLC is further sub classified based on histological features into squamous cell carcinoma, adenocarcinoma, large cell carcinoma, neuroendocrine tumours and carcinomas with sarcomatous elements.

NSCLC can now be subdivided into different molecular groups with different treatment implications with the standard of care for patients with advanced-stage NSCLC shifting from selecting therapy empirically based on a patient’s clinicopathologic features to using biomarker-driven treatment algorithms based on the molecular profile of a patient’s tumour [10,11]. Genetic alterations with emerging targeted agents include BRAF V600 mutations, MET amplification, ROS1 rearrangements, HER2 mutations and RET rearrangements [12,13].

Histomorphologic diagnosis has traditionally been used to select therapy based on the clinicopathologic factors to select available drugs for an individual patient [11]. This approach has been replaced by the advancement of personalised medicine. The majority of current personalised medicine using targeted therapy, is based on the use of single gene-based molecular tests such as EGFR to select specific drugs for an individual patient [10]. Traditionally further developments would undergo an evolutionary process with an incremental increase in personalised medicine with the use of multiplexed molecular tests with increased sensitivity and outputs for the therapeutically effective selection of available drugs for an individual patient [11]. However, due to rapid advancements in technology, personalised medicine using an integrated genomic profile from high-throughput next-generation sequencing to tailor targeted treatment for an individual patient can be used now.

The National Comprehensive Cancer Network (NCCN) has updated the Guidelines for Clinical Practice Guidelines Non-Small Cell Lung Cancer Version 1.2015. For metastatic disease: “The NCCN NSCLC Guidelines Panel strongly endorses broader molecular profiling with the goal of identifying rare driver mutations for which effective drugs may already be available, or to appropriately counsel patients regarding the availability of clinical trials. Broad molecular profiling is a key component of the improvement of care of patients with NSCLC [12].”.

In Australia, EGFR molecular testing is subsidied for testing of tumour tissue from a patient diagnosed with non-small cell lung cancer, shown to have non-squamous histology or histology not otherwise specified to determine EGFR gene status for access to erlotinib or gefitinib.

The epidermal growth factor receptor, EGFR, is a cellular transmembrane receptor. It is expressed in a number of different tumour types including non-small cell lung cancer (NSCLC). Some mutations in the gene (Exons 18–21) result in activation of the gene without ligand binding, resulting in cellular proliferation. Clinical studies have found that the prevalence of EGFR activating mutations in NSCLC is approximately 20%–40% in Asians and 10% among Caucasians [14]. The two most common mutations—in-frame deletions in exon 19 and L858R point mutations in exon 21—account for 90% of all EGFR mutations [15]. The determination of EGFR mutation status is critical when evaluating a patient for anti-EGFR targeted therapy. EGFR tyrosine kinase inhibitors have a high affinity for mutant EGFR and block the downstream effects of activation resulting in inhibition of tumour growth and cell survival [14].

Compared to lung cancers with EGFR activating mutations, tumours with no mutation detected in EGFR are less sensitive to the EGFR TKIs, erlotinib and gefitinib.

Patients given first generation TKIs will usually develop resistance and the EGFR p.T790M mutation accounts for approximately 49%–63% of acquired resistance to first-generation EGFR TKIs such as erlotinib and gefitinib [16]. *De novo* EGFR p.T790M mutations have also been reported in NSCLC and are usually found in combination with another EGFR mutation. A primary EGFR p.T790M mutation is present in <1% of somatic mutations and may also occur as a germline mutation [17,18,19,20]. The p. T790M mutation impairs binding of erlotinib and gefitinib resulting in primary resistance. Second generation TKIs (dacomitinib and afatinib) are irreversible TKIs with activity against tumours with the T790M mutation. Third generation TKIs target EGFR mutations including tumours with the p. T790M mutation, but not wild-type EGFR (AZD9291, CO-1686 and HM61713) [20,21].

Rearrangements of the receptor tyrosine kinase *ALK* also occur in approximately 4% of NSCLC. The fusion partners include *EML4*, *KIF5B*, *TFG* and *KLC-1*. These fusion genes result in activation of ALK which is linked to cell proliferation and inhibition of apoptosis mediated through the RAS/RAF/MAPK1, PI3K/AKT and JAK3-STAT3 signalling pathways [22]. *EGFR* mutations and *ALK* fusion mutations are usually mutually exclusive, however dual mutations have been reported [23]. Tumours with *ALK* fusion mutations are responsive to the tyrosine kinase inhibitor crizotinib however, as with *EGFR* mutations and TKIs, resistance develops with evidence of secondary *ALK* point mutations and activation of *EGFR* signalling in some cases [22].

## 5. Colorectal Carcinoma

KRAS is part of the RAS family of proto-oncogenes and encodes a G-protein with a critical role in cell signalling pathways. Activating mutations lead to increased signalling through multiple downstream growth promoting pathways. Mutations in the KRAS gene occur in multiple tumour types including colorectal carcinoma, non-small cell lung cancer and pancreatic carcinoma. Some mutations in the KRAS gene (approximately 40% of colorectal carcinomas) and the NRAS gene (approximately 1%–6%) are associated with poor prognosis and a lack of response to anti-EGFR antibody therapy such as cetuximab and panitumumab [23,24,25,26,27,28,29,30,31,32].

## 6. Technology

Tumour biopsy remains the standard of care for diagnosis of cancer and improvements in imaging technology have resulted in a significant improvement in cancer diagnosis. Currently the standard approach for cancer diagnosis is the examination of tumour tissue through either removing cells through a small needle (fine needle aspiration cytology), or histological examination of a biopsy or surgical excision specimen. However, there are limitations to both of these methods. These procedures are invasive and involve some risk to the patient. Tumours may occur in tissues that are difficult to access and the size of the lesion may limit the ability to sample tissue adequately. Metastatic lesions are often not resampled and changes between the primary tumour and metastatic tumour may occur that impact on response to treatment. Tumour heterogeneity also poses problems with differences in molecular characteristics between different areas of a single tumour.

The molecular diagnosis of DNA mutations in fixed tumour tissue poses significant challenges. The majority of diagnostic material is formalin-fixed paraffin embedded tissue (FFPE). Fixation of tissues with formalin results in a number of chemical reactions including DNA denaturation, fragmentation of DNA and the introduction of nonreporducible sequence alterations [33]. The majority of artifactual SNVs in FFPE DNA are transitional C:G > T:A changes resulting from cytosine deamination to uracil. The number of detected sequence artifacts varies across FFPE samples. C:G > T:A SNVs often account for 50% of all artifactual SNVs in FFPE DNA samples [34].

Fixation of samples using formalin at room temperature results in poor preservation of high-molecular weight DNA with the size of the extracted DNA being directly related to the fixation temperature. Up to 30% of nucleic acids may be lost during fixation [35,36]. Kapp *et al.* reviewed the processing of FFPE samples and noted that the mutation detection failure was 11.9% with 80% of these attributable to pre-PCR error [37].

**Figure 1:**
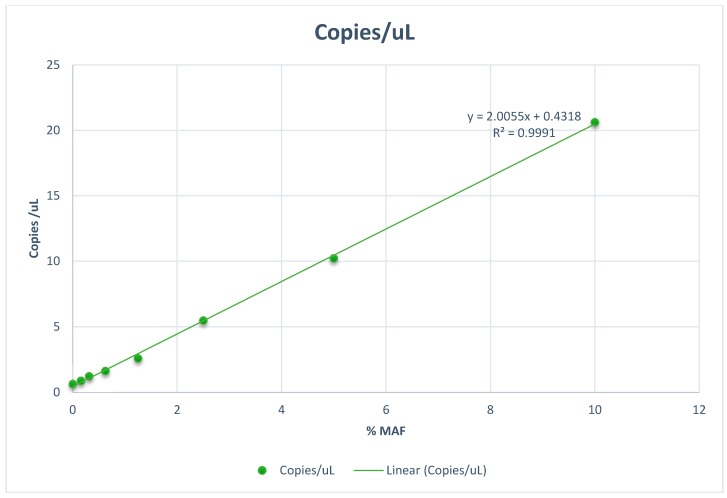
Limit of detection curve for AKT1 c.49G>A p.E17K using combined allele specific PCR and digital PCR.

Advances in technology have resulted in the development of new techniques including novel sequencing technologies, massive parallel sequencing, highly sensitive quantitative polymerase chain reaction (PCR) testing, digital PCR (Figure 1) and BEAMing (beads, emulsions, amplification and magnetics) digital PCR [38]. Enrichment techniques such as synchronous thermal-electrophoretic separation [39] can also be used to improve detection limits (Table 1).

**Table 1:** Technologies utilised for detection of ctDNA in plasma and examples of use in oncology.

Technology	Sensitivity	Tumour Type	Gene/Mutations	Clinical Utility	References
Droplet Digital PCR (ddPCR)	~0.001%–0.03%	NSCLC	*EGFR* T790M	Treatment Decision, Resistance	[40,41]
	0.04%	NSCLC	*EGFR*	Treatment Decision	[42]
	0.01%	Breast	*PIK3CA*	Prognosis	[43]
	0.1%	CRC	*KRAS*	Treatment Decision	[40]
	0.005%	Melanoma	*BRAF* V600E	Tumour Burden	[41]
COLD-ddPCR	0.2%–1.2%	Multiple	*EGFR*, *TP53*	Monitoring	[44]
Nanofluidic Digital PCR	0.05%	NSCLC	*EGFR* T790M	Treatment Decision, Resistance	[45]
BEAMing Digital PCR	0.01%	Breast	*PIK3CA*	Treatment Decision	[46]
castPCR	0.1%–1%	Ovarian	Beta-globin	Prognosis	[47]
ARMS-PCR	~1%	CRC	*KRAS*, *BRAF*	Prognosis	[48,49]
		Breast	*PIK3CA*	Treatment Decision	[50]
Clamping PCR	0.1%–1%	CRC	*KRAS*	Treatment Decision, Prognosis	[51,52]
		NSCLC	*EGFR*	Diagnosis	[53]
Synchronous TES	0.01%	NSCLC	*EGFR T790M*	Treatment Decision, Resistance	[54]
NGS	<5%	Ovarian	*TP53*	Tumour Burden	[55]
	0.5%	Breast	Multiple	Treatment Decision	[56]

CRC—Colorectal carcinoma; ARMS—Amplification Refractory Mutation System; castPCR—Competitive Allele Specific TaqMan PCR; TES—thermal-electrophoretic separation.

### 6.1. Digital PCR

Digital PCR uses a limiting dilution of DNA into individual PCR reactions. There are two methods of performing the partitioning of the individual reactions: nanofluidics with well based partitioning (Fluidigm and Thermo Fisher OpenArray and QuantStudio) and microdroplet partitioning using a water-in-oil emulsion (RainDance Technologies and Biorad). The advantages of digital PCR are that it is more precise, less susceptible to inhibition and is quantitative [57] with a linear response to the number of DNA copies present. This eliminates the requirement for a standard curve to be performed and enables the use of digital PCR to perform copy number variation assays for specific genes such as *ERBB2* (HER2).

Whilst standard PCR reactions can be used for digital PCR, the assay can also use allele specific PCR reactions to selectively identify mutations present in low percentages.

### 6.2. BEAMing

BEAMing (beads, emulsions, amplification and magnetics) is a process built on emulsion PCR where each compartment contains a bead that is coated with thousands of copies of the single DNA molecule originally present. Millions of beads are analysed using flow cytometry [58]. This assay can be multiplexed for detection of a number of different mutations simultaneously.

### 6.3. Synchronous Thermal-Electrophoretic Separation

Short oligonucleotide probes complementary to the targets are covalently linked to the separation matrix, a denatured sample is injected into the gel and the gel is held at the melting temperature of the target-probe duplex and the DNA is separated by alternating electric fields. This process enriches for target alleles whilst wild-type alleles are removed [39]. This methodology can also be multiplexed for specific gene mutations.

These advances have improved the detection sensitivity of DNA and RNA molecules enabling the use of alternate samples as the source of biomarker analysis.

## 7. Cell-Free DNA

One advantage of using molecular biomarkers and particularly DNA somatic mutations, is that these mutations are only present in tumour cell DNA and are not present in normal cells. This provides an extremely specific biomarker for cancer that can be detected and tracked over time.

Cell-free DNA (cfDNA) is released from normal cells and tumours by programmed cell death (apoptosis) and comprises small fragments of nucleic acid that are not associated with cells or cell fragments. This cfDNA is present in the plasma of blood in all people.

Detection of cfDNA mutant DNA (cftNA) was described in 1994 [59,60] and there have been multiple publications in the last two decades [3,40,46,49,61,62,63,64,65,66,67]. cfDNA fragments in blood measure between 150 to 200 base pairs in length [68] and ctDNA comprises between 0.01% to 90% of cfDNA [69].

Tumours containing ~50 million malignant cells release sufficient DNA for the detection of circulating cell-free tumour DNA (ctDNA) in blood [70] and this is below the limit of resolution of radiology studies. For imaging studies, there are still limitations to the resolution and difficulties distinguishing benign from malignant lesions particularly for small lesions [71,72]. Radiological imaging studies are only able to detect tumours when they are approximately 7–10 mm in size and contain ~1 billion cells. As the volume of the tumour increases, the number of apoptotic and dead cells increases due to increased cellular turnover.

ctDNA can be detected and monitored using specific assays for tumour and patient specific mutations or by using *de novo* sequencing looking for a broader range of mutations [56]. Massive parallel sequencing (MPS) has recently been used to detect ctDNA as it does not require knowledge of a specific mutation in the primary tumour [3]. Rothe *et al.* [56] analysed samples from 69 breast cancer tumour samples and 31 plasma samples using semiconductor sequencing for 50 cancer genes covering 2800 COSMIC mutations. Identical mutations in the tumour and plasma were identified in nine patients; in two patients, a mutation was identified in tumour but not in plasma; in two patients, a mutation was identified in plasma but not in the tumour. Seventy-six percent (13 of 17) of patients showed concordant results between tumour and whereas in the remaining four (24%) patients, the results were discordant, providing complementary information. Ongoing clinical trials will provide more information on the clinical utility of this approach [3].

Circulating cell-free foetal DNA is present within maternal plasma. Isolation and sequencing of this foetal component has entered clinical practice as non-invasive prenatal screening (NIPT) for trisomy 21, trisomy 18 and trisomy 13 as well as detection of microdeletion syndromes and foetal sex in some assays [73]. The overall sensitivity and specificity of NIPT is close to 99% [74], however in a small number of cases there have been discordant results identified between NIPT and the invasive diagnostic tests. Investigation of these cases has identified patients where metastatic disease appears to account for the discordant results [75]. In one patient metastatic small cell carcinoma of the vagina was diagnosed postpartum and this was postulated to account for the aneuploidies of chromosome 13 and 18 that were identified on NIPT [75]. In another report, massive parallel sequencing was used to detect genomic imbalances for NIPT and identified a patient with Hodgkins Disease [76,77]. In 2014 about 800,000 women had NIPT for detection of aneuploidies and 26 of these patients were also identified as having cancer [78]. NIPT, whilst more sensitive and specific than current aneuploidy screening assays, is still a screening test. Discordant results should be investigated as in a small proportion of patients the discrepancy will be due to maternal malignancy. Other causes of discordant results include placental mosaicism and maternal copy number variation.

## 8. Applications

Circulating tumour DNA fragments (ctDNA) contain identical genetic defects to those seen in the primary tumour itself [79]. Because ctDNA fragments are released from all parts of the tumour the ctDNA is in fact a liquid biopsy [79]. The main aim of ctDNA utilisation is to supplement or replace tissue biopsies. The assay is minimally invasive and can be repeated easily at multiple time points to enable close monitoring of patient response to treatment or early identification or relapse. The capacity of mutant ctDNA to reflect changes in the tumour can be applied to a number of different areas in oncology. The first application and perhaps the easiest to implement is the use of ctDNA as a new biomarker, similar to protein biomarkers already in use, to monitor tumour burden. Other applications include the detection of minimal residual disease, the early detection of therapy resistance, the assessment of molecular heterogeneity and the early detection of disease.

The clinical utility of the molecular analysis of tissue biopsies has been established and as detailed above there are specific gene mutations that correlate with the response to targeted therapy. ctDNA analysis has been compared to the tissue molecular diagnostics across a variety of tumour types in a number of different applications. These comparisons generally used specific targeted assays designed for each tumour type and each patient for enhanced sensitivity, rather than agnostic methodologies such as exome sequencing.

In the Phase II SHIVA trial, ctDNA was evaluated in 34 patients covering 18 different tumour types including mutations in 46 genes with a multiplexed next-generation sequencing panel [80]. In 27 patients, 28 of 29 mutations identified in metastasis biopsies (97%) were detected in matched ctDNA. Mutation detection in seven other patients failed using metastatic tissue biopsy samples due to inadequate biopsy material, but was successful in all plasma DNA samples. The results from this trial suggest that ctDNA analysis is a potential alternative and/or replacement to conventional tissue analysis irrespective of cancer type and metastatic site. In addition, ctDNA can be used where tissue biopsies fail or cannot be performed due to inaccessibility. ctDNA can then be used for multiplexed mutation detection in selecting personalized therapies based on the patient’s tumour genetic content [81] similar to the molecular assays currently performed on tumour tissue samples [80].

### 8.1. Monitoring of Tumour Burden

The monitoring of disease burden can be problematic. Following treatment, radiological changes may not reflect true residual tumour burden as it may be difficult to distinguish tumour from surrounding tissue reactions. Repeat biopsies for histological confirmation of residual tumour and even fine needle aspirates may also be difficult to obtain in the context of limited disease and may not be representative of each individual tumour site due to sampling variation. For this reason blood biomarkers are used when available to monitor tumour burden following treatment. Many malignancies do not have a reliable protein biomarker and the biomarkers are often not specific for cancer. As cfDNA has a short half-life in blood from approximately 15 min [81] to several hours [82] changes in the tumour can be evaluated in days rather than weeks [82]. Changes in ctDNA can often occur prior to changes seen in imaging studies [61,83] and ctDNA is exquisitely specific for an individual’s tumour. Patient-specific somatic structural variants have been used to monitor disease burden following colorectal cancer surgery [84]. Droplet digital PCR was used for 151 serial plasma samples from six relapsing and five non-relapsing colorectal cancer patients. The patient specific assays were a specific and highly sensitive approach for monitoring of the disease load [84].

Similar results have been seen in patients with melanoma with real-time monitoring of tumour burden [41,85]. Twelve patients undergoing treatment with immune therapy blockade were tracked using specific ctDNA hotspot mutations. ctDNA levels correlated with clinical and radiological outcomes and in one patient preceded eventual tumour progression [85]. In the second study, 20 patients with *BRAF* V600E mutation were monitored during treatment with BRAF inhibitors with an increase in the concentration of cfBRAF (V600E) at disease progression [41].

QIAGEN’s circulating tumour plasma DNA test for EGFR mutations in NSCLC received CE-IVD status in January 2015. In February 2015, the China Food and Drug Administration (CFDA) approved an update to the gefitinib label for advanced NSCLC to include blood based diagnostics when tumour tissue is not evaluable.

### 8.2. Monitoring of Minimal Residual Disease

In the case of curative surgery for cancer, we currently have no means to identify patients who are cured from those patients that may still have residual disease. Currently predicting which patients are disease free is based predominantly on clinical and pathology criteria. Circulating tumour cells (CTCs) have been used to monitor patients with specific cancer subtypes and the number of CTCs has shown a correlation with prognosis for breast, prostate and lung cancer [86]. In a comparison of CTCs and ctDNA in metastatic breast cancer, ctDNA levels showed a greater dynamic range and greater correlation with changes in tumour burden compared to CTCs [61]. In this study, ctDNA also provided the earliest measure of treatment response in 10 of 19 women (53%) [61].

ctDNA is a potential marker of residual disease after surgery and should be measured after the surgery but before the commencement of adjuvant therapy (generally 6–8 weeks after surgery) [79].

The changes of ctDNA in response to treatment is consistent across all tumour types’ studies so far. Levels of ctDNA correspond with the clinical course and ctDNA increases with disease progression and correspondingly decreases with response to therapy. In breast cancer ctDNA correlates with changes in tumour burden [61]. In this study, ctDNA was detected in 97% of patients with metastatic breast cancer in whom somatic mutations in the tumour were detected compared to CA15-3 being detected in only in 78% and circulating tumour cells in 87%. The dynamic range for ctDNA was greater compared to the other two biomarkers and there was a better correlation with changes in the tumour burden [61]. The use of ctDNA has also been used in early breast cancer in a small prospective trial of 20 patients to assess recurrence after neoadjuvant therapy [87]. The primary tumour was analysed using next generation sequencing (NGS) and mutations were identified in 60% of the tumours. Personalized tumour specific digital PCR assays were then used to track the ctDNA over time. ctDNA was detected in 75% (9/12) of samples at baseline. Five patients relapsed at a median of 8.1 (5–16.6) months post-surgery, with the remaining patients disease free at a median of 11.5 months post-surgery. Four of patients who relapsed had specific ctDNA detectable in the first six months post-surgery, and all patients with detectable ctDNA relapsed. None of the patients who had not relapsed had detectable ctDNA post-surgery (*p* = 0.01), indicating clearance by the primary treatment. A patient with isolated relapse in the brain on trastuzumab, after a pathological complete response in the primary, did not have detectable ctDNA at relapse [87].

Similar changes have been identified in colorectal carcinoma [82] with ctDNA being evaluated in patients undergoing multimodality therapy for colorectal cancer. A group pf patients were followed for 2–5 years after treatment. ctDNA was able to detect minimal residual disease after surgery [82]. Relapse occurred in virtually all patients with detectable ctDNA post-treatment whereas relapse did not occur in the four patients where ctDNA was undetectable at the first follow-up visit [82].

Monitoring of ctDNA for residual disease has been used in patients with melanoma and lung cancer [55,88,89,90]. Specific droplet digital PCR assays were used for serial plasma genotyping of EGFR-mutant lung cancer in patients being treated with erlotinib [91]. *EGFR* mutations were detected prior to treatment with complete plasma response demonstrated in most cases [92]. Increasing levels of *EGFR* T790M were identified before objective disease progression [91].

In gastric cancer targeted deep sequencing of *TP53* mutations in plasma cell-free DNA was used to monitor carcinoma progression and residual disease [92]. Three of ten patients had detectable TP53 ctDNA mutations preoperatively and the ctDNA fraction correlated with the disease status [92].

### 8.3. Monitoring of Tumour Heterogeneity

Some level of heterogeneity exists in all tumours. Tumours can have intratumoural diversity with populations of cells showing clonal evolution and different combinations of mutations [93,94].

This is well known in anatomical pathology where histopathologists commonly observe morphological variation in tumours. Similarly, variation in morphological biomarkers is well known. Expression of oestrogen receptor in breast cancer using immunohistochemistry shows different tumours express different percentages and different intensity of staining across a tumour. *ERBB2* (HER2) staining can also show clonal variation in breast carcinomas and this is more pronounced in gastric adenocarcinomas with variation in both HER2 protein expression and gene amplification occurring across a single tumour [95].

International projects have sequenced large numbers of primary tumours and established the degree of molecular heterogeneity seen in primary tumours across the broad spectrum of human cancers [96]. The Cancer Genome Atlas (TCGA) has analysed 12 different tumour types and identified a total of 127 driver genes, with two to six driver gene mutations on average per individual tumour [97]. Sequencing studies of breast carcinoma cohorts have also shown that point mutations evolve gradually over time resulting in extensive clonal diversity [94]. Tumour heterogeneity also exists between primary tumours and metastatic sites [96] and mutational heterogeneity has been identified between tumour tissue and circulating tumour cells in colorectal carcinoma [98,99].

ctDNA analysis can provide an overview of all the cells in a patient’s tumour simultaneously and this takes into account variations in different cells in the tumour and may provide an early indication if cells are becoming resistant to therapy. Massive parallel sequencing (MPS) has been used as a proof-of-concept in a patient with breast carcinoma to demonstrate that high-depth targeted MPS of plasma-derived ctDNA can be utilised for *de novo* mutation identification and monitoring of somatic genetic alterations during the course of targeted therapy [99]. This approach may be able to be used to overcome the challenges posed by intra-tumour genetic heterogeneity [99].

In addition, mutations in different pathways in the tumour cells can be used to plan combination treatment and this approach can prevent resistance developing in the tumour cells and improve response [100].

### 8.4. Monitoring of Molecular Resistance

Resistance to treatment results from the acquisition of new molecular changes in the cancer cells [101,102,103]. Profiling of tumours enables the identification of genomic mutations in the cancers, however, depending on the healthcare system, this may only be used for single genes. Wider genomic profiling identifies more targets in the tumour samples from each patient. Targeted therapy is then used to treat patients where the tumour has specific mutations (or lack of mutations for RAS in colorectal carcinoma) and this has resulted in modest anti-tumour effects [102]. Tumour heterogeneity results in the emergence of resistant clones [101,102,103] and ctDNA or liquid biopsies can be used to monitor the development of resistance to therapy during treatment. This has been demonstrated for leukaemia, lung cancer, bowel cancer and malignant melanoma [79,103].

This understanding of the mechanisms of resistance can be used to plan combination treatment and institute alternate therapies.

### 8.5. Early Diagnosis of Tumours

In a prospective study, 29 patients with primary breast carcinomas were analysed for *PIK3CA* mutations using droplet digital PCR [104]. Of the 15 *PIK3CA* mutations detected in tumours by droplet digital PCR, 14 of the corresponding ctDNA mutations were detected in presurgical plasma, whereas no mutations were found in plasma from patients with *PIK3CA* wild-type tumours (sensitivity 93.3%, specificity 100%). Ten patients with ctDNA detected presurgery had analysis of postsurgery plasma, with five patients having detectable ctDNA postsurgery [104].

More than 90% of pancreatic adenocarcinomas have *KRAS* mutations. *KRAS* mutations in ctDNA have been analysed in 66 consecutive patients with pancreatic cancer [105]. The *KRAS* mutation rate in ctDNA was 62.6%. Analysis of ctDNA can be used for detecting mutations in patients with pancreatic cancer and this method may have potential as a new strategy for the diagnosis of pancreatic cancer as well as for predicting survival [105].

More work is needed to develop these new assays for the early detection of cancer, however these approaches, which are very specific and are increasing in sensitivity, open up the possibility of using ctDNA for screening in the future.

## 9. Conclusions

Traditionally, the diagnosis of cancers has been based on histology, the morphological features identified on routine haematoxylin and eosin (H & E) staining and the site of origin. Immunohistochemistry was implemented into routine diagnostic practice in the 1970s and provides supplementary information to the routine H & E stain for tumour diagnosis. Despite these advances, a tissue sample is required necessitating a biopsy, excision or another invasive procedure such as fine needle aspiration. The majority of samples used for diagnosis are formalin fixed, paraffin embedded samples. FFPE samples are problematic for molecular testing, particularly for utilisation of DNA and RNA. Whilst alternate fixatives are available, these have not been implemented into routine diagnostic practice for a number of reasons including cost, specialised collection procedures, specialised processing of samples, poor morphology compared to FFPE and significant disruption to routine workflows.

Monitoring of a tumour response or confirmation of metastatic disease is often based on radiological studies and whilst there is some move towards the biopsy of possible metastatic deposits, this is not regarded as routine practice. Tracking of tumour response to therapy by repeat biopsies in the case of neoadjuvant treatment is also not regarded favourably.

Alternate methodologies have been pursued for the identification of minimally invasive testing for biomarkers that can provide the information required to monitor and diagnose cancer without the use of FFPE tissue samples. This challenge appears to be reaching fruition with the combination of molecular testing to identify specific mutations only occurring in cancer, the recognition of the presence of these mutations in circulating DNA in blood and the development of highly sensitive technologies to detect this material.

The combination of these techniques has now enabled the detection of circulating cell-free mutant DNA on blood samples and patients with tumours can now be monitored over time to assist in the assessment of response to treatment. The testing is specific to each individual’s tumour and forms part of personalised medicine.

The evaluation of the clinical utility of these tests, defined as the measure of net health benefits, is on-going.
